# Inhibition of G protein-gated *K*^*+*^ channels by tertiapin-Q rescues sinus node dysfunction and atrioventricular conduction in mouse models of primary bradycardia

**DOI:** 10.1038/s41598-020-66673-8

**Published:** 2020-06-17

**Authors:** Isabelle Bidaud, Antony Chung You Chong, Agnes Carcouet, Stephan De Waard, Flavien Charpentier, Michel Ronjat, Michel De Waard, Dirk Isbrandt, Kevin Wickman, Anne Vincent, Matteo E. Mangoni, Pietro Mesirca

**Affiliations:** 1 IGF, Univ. Montpellier, CNRS, INSERM, Montpellier, France; 2 LabEx Ion Channels Science and Therapeutics (ICST), Montpellier, France; 3 l’institut du thorax, INSERM, CNRS, UNIV Nantes, F-44007 Nantes, France; 4 l’institut du thorax, CHU Nantes, Nantes, France; 5 Institute for Molecular and Behavioural Neuroscience, University of Cologne, 50937 Cologne, Germany; 6 Experimental Neurophysiology, German Centre for Neurodegenerative Diseases (DZNE), 53175 Bonn, Germany; 7 Department of Pharmacology, University of Minnesota, Minneapolis, USA

**Keywords:** Arrhythmias, Cardiomyopathies

## Abstract

Sinus node (SAN) dysfunction (SND) manifests as low heart rate (HR) and is often accompanied by atrial tachycardia or atrioventricular (AV) block. The only currently available therapy for chronic SND is the implantation of an electronic pacemaker. Because of the growing burden of SND in the population, new pharmacological therapies of chronic SND and heart block are desirable. We developed a collection of genetically modified mouse strains recapitulating human primary SND associated with different degrees of AV block. These mice were generated with genetic ablation of L-type Ca_v_1.3 (Ca_v_1.3^−/−^), T-type Ca_v_3.1 (Ca_v_3.1^−/−^), or both (Ca_v_1.3^−/−^/Ca_v_3.1^−/−^). We also studied mice haplo-insufficient for the Na^+^ channel Na_v_1.5 (Na_v_1.5^+/^) and mice in which the cAMP-dependent regulation of hyperpolarization-activated f-(HCN4) channels has been abolished (HCN4-CNBD). We analysed, by telemetric ECG recording, whether pharmacological inhibition of the G-protein-activated K^+^ current (*I*_*KACh*_) by the peptide tertiapin-Q could improve HR and AV conduction in these mouse strains. Tertiapin-Q significantly improved the HR of Ca_v_1.3^−/−^ (19%), Ca_v_1.3^−/−^/Ca_v_3.1^−/−^ (23%) and HCN4-CNBD (14%) mice. Tertiapin-Q also improved cardiac conduction of Na_v_1.5^+/−^ mice by 24%. Our data suggest that the development of pharmacological *I*_*KACh*_ inhibitors for the management of SND and conduction disease is a viable approach.

## Introduction

The pacemaker activity of the sino-atrial node (SAN) generates heart automaticity and controls the heart rate in everyday life. Sinus node dysfunction (SND), also referred to as ‘sick sinus syndrome’, is caused by failure to generate a normal SAN impulse^1^. The diagnosis of SND is based on the correlation between the patient’s symptoms and ECG hallmarks (*e.g*., SAN bradycardia), which provide important criteria for proceeding to permanent pacemaking (PPM) by an implanted device^2,3^. When unrelated to sports/physical training or other physiological conditions, sinus bradycardia is generally defined as a regular heart rate below 50 beats per minute^2^. SND patients are diagnosed as having one or more of the following ECG signs: (1) persistent, unexpected sinus bradycardia, (2) short periods of sinus arrest during which atrial or junctional rhythms replace normal sinus rhythm, (3) long periods of sinus arrest in the absence of junctional rhythms, resulting in cardiac arrest, (4) chronic atrial fibrillation, concomitant with ineffectiveness of cardioversion to restore normal sinus rhythm, and finally (5) episodes of sinus exit block not related to drug therapy^4^. Sinus pauses, or sinus arrest, are included in the current definition of SND, particularly when they manifest as ‘tachycardia-bradycardia syndromes’ during which sinus or ectopic bradycardia, sinus pauses, or sinus arrest, follow periods of abnormal atrial tachycardia, atrial fibrillation, or flutter^5^. Another hallmark of SND is chronotropic incompetence, defined as the inability of the heart rate to attain 80% of the expected heart rate reserve during exercise. In addition, conditions resulting from long-term physical training and increased vagal tone can lead to relevant SND in early or later stages of life^6^. Diseases of cardiac automaticity and SND are a growing clinical problem. In Europe and in the U.S., SND necessitates the implantation of nearly half a million pacemaker devices each year and is predicted to double over the next half century, particularly in the aging population^7^. Not only does SND affect the aging population, but age-associated pathologies potentiate SND. For example, the intrinsic, age-related SND has clinical manifestations that are accelerated by heart failure^8^ and diabetes^9^. These pathologies are independent risk factors for increased mortality and are associated with worsened patient outcomes^10–12^. Pharmacological treatment of SND using atropine, theophylline, or isoproterenol is indicated in patients with acute SND. However, these molecules present severe extracardiac side effects preventing long-term usage^2^. Moreover, most patients have chronic and irreversible symptoms that can only be treated with PPM^2,3^. Considering the increasing global impact of SND in the general population, it is generally agreed that new pharmacological strategies offering therapeutic options complementary to PPM are needed.

Several forms and causes of SND are known. In principle, primary (idiopathic) forms, caused by genetic inheritance^13,14^ and secondary forms, occurring as comorbidities of cardiovascular or systemic pathology (*e.g*., cardiac ischemia, heart failure, atrial fibrillation, diabetes)^7^ are known. Among the primary SND forms, loss-of-function in ion channels underlying SAN pacemaker activity and atrioventricular (AV) conduction are major causes of SND^13,14^. These primary forms of SND are thus interesting models for testing new potential therapeutic strategies designed to control pathologies of cardiac automaticity.

We developed a collection of mouse models of human primary SND induced by loss-of-function in ion channels. This collection includes mice with genetic ablation of L-type Ca_v_1.3^15^, or T-type Ca_v_3.1^16,17^ voltage-gated Ca^2+^ channels (VGCCs), as well as mice conditionally expressing hyperpolarization-activated f-(HCN4) channels devoid of cAMP-dependent regulation of channel activation^18^. These models faithfully recapitulate a wide range of human primary SND hallmarks, from moderate sinus bradycardia^19,20^ to SND associated with severe AV block^21–23^ and atrial tachycardia^24^. Furthermore, we explored the phenotype of mice haplo-insufficient for cardiac voltage-dependent Na^+^ channel Na_V_1.5^25–27^. Na_v_1.5^+/−^ mice recapitulate Lev-Lènegre syndrome^28^, which is characterized by progressive AV block and intraventricular conduction dysfunction,

In a previous study, we showed that genetic ablation of *I*_*KACh*_ prevents SND in mice deficient in L-type Ca_v_1.3 channels^29^. However, it is unknown whether pharmacological inhibition of *I*_*KACh*_ by tertiapin-Q can prevent SND and AV dysfunction in a wide set of mouse models of SND exhibiting loss-of-function of ion channels involved in SAN pacemaking and AV conduction. Tertiapin is a 21-residue peptide extracted from the honey bee venom^30^, which potently blocks G protein-activated K^+^ (GIRK1/GIRK4) channels^31,32^ mediating the cardiac *I*_*KACh*_ current^33^. Blockade of *I*_*KACh*_ by tertiapin reduces the negative chronotropic and dromotropic effects of acetylcholine in isolated rabbit and guinea pig hearts^34^. Here, we show that pharmacological inhibition of the cardiac *I*_*KACh*_ by tertiapin-Q, a synthetic stabilized analog form of tertiapin^35^, improves heart rate and normalizes AV conduction in mouse models of SND.

## Results

### Mouse models with genetic ablation of voltage-gated Ca^2+^ channels recapitulate hallmarks of SND syndrome and AV dysfunction

**Figure 1:**
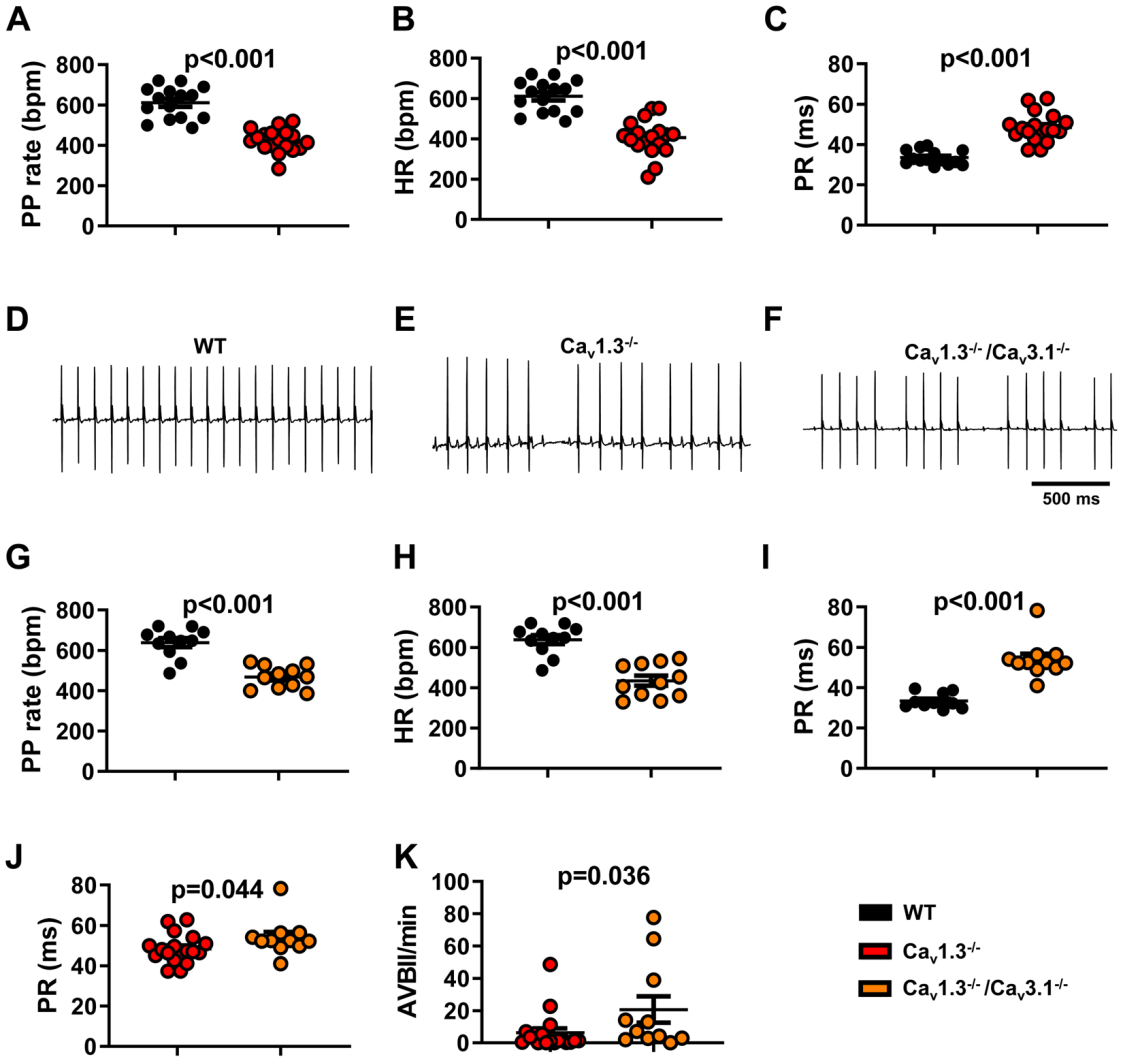
PP rate, heart rate and atrioventricular conduction in Ca_v_1.3^−/−^ and Ca_v_1.3^−/−^/Ca_v_3.1^−/−^ mice under basal conditions. PP rate, heart rate and PR interval recorded in WT (black circle), Ca_v_1.3^−/−^ (red circle, (**A–C**) and Ca_v_1.3^−/−^/Ca_v_3.1^−/−^ mice (orange circle, **G**-**I**), together with corresponding representative samples of telemetric ECG recordings (WT in (**D**), Ca_v_1.3^−/−^ in (**E**) and Ca_v_1.3^−/−^/Ca_v_3.1^−/−^ in (**F**). (**J**) PR interval recorded in Ca_v_1.3^−/−^ and Ca_v_1.3^−/−^/Ca_v_3.1^−/−^ mice during 24 h telemetric recording. Statistics: unpaired t test. (**K**) Number of 2^nd^-degree atrioventricular blocks per minutes (AVBII/min) in Ca_v_1.3^−/−^ and Ca_v_1.3^−/−^/Ca_v_3.1^−/−^ mutant animals. Statistics: Mann-Whitney test. Error bars define the s.e.m.

We first compared the PP rate (from PP intervals, reflecting the SAN rate), the heart rate (from RR intervals) and the AV conduction time (PR intervals) under basal conditions recorded from mouse strains carrying genetic ablation of VGCCs. Animals deficient in L-type Ca_v_1.3 channel (Ca_v_1.3^−/−^) displayed slower PP rate (424 ± 13 bpm, n = 18), a reduced mean 24-h heart rate (406 ± 21 bpm, n = 18) and a prolonged PR interval (48 ± 2 ms, n = 18) in comparison to their wild-type (WT) counterparts (611 ± 20 bpm, n = 15, and 33 ± 1 ms, n = 14; Fig. 1A–E), which was associated with a high incidence of second-degree AV blocks (AVBII, 6 ± 3 AVBII/min, n = 18) that were absent in WT animals. Genetic ablation of T-type Ca_v_3.1 channels, slightly, but significantly, affected the basal heart rate (608 ± 7 bpm, in WT animals, *vs* 571 ± 12 bpm, in mutants; Supplementary Fig. 1A) and induced a moderate prolongation of the PR interval in comparison to WT animals (34.5 ± 1 ms *vs* 32.5 ± 1 ms, respectively; Supplementary Fig. 1B). Concomitant loss of Ca_v_1.3 and Ca_v_3.1 channels decreased the PP rate (468 ± 17 bpm, n = 11, Fig. 1G) and induced a strong heart rate reduction (435 ± 25 bpm, n = 11; Fig. 1F,H) in mutant (Ca_v_1.3^−/−^/Ca_v_3.1^−/−^) mice, which was quantitatively similar to that recorded in Ca_v_1.3^−/−^ mice (406 ± 21 bpm; Supplementary Fig. 2). The concomitant ablation of Ca_v_1.3 and Ca_v_3.1 resulted in an additive effect on AV conduction time. Indeed, the PR interval (54 ± 3 ms, n = 11; Fig. 1I,J) and the number of AVBII episodes (21 ± 8 AVBII/min, n = 11) were increased in Ca_v_1.3^−/−^/Ca_v_3.1^−/−^ in comparison to Ca_v_1.3^−/−^ (Fig. 1J,K). These results are consistent with our previously published data showing SND associated with AV block and dysfunction in Ca_v_1.3^−/−^^29^, Ca_v_3.1^−/−^^16^ and Ca_v_1.3^−/−^/Ca_v_3.1^−/−^ mice^17^.

### SND hallmarks in mice with loss-of-function of f-(HCN4) channels and AV conduction dysfunction in mice deficient in voltage-dependent cardiac Na^+^ (Na_v_1.5) channels

**Figure 2:**
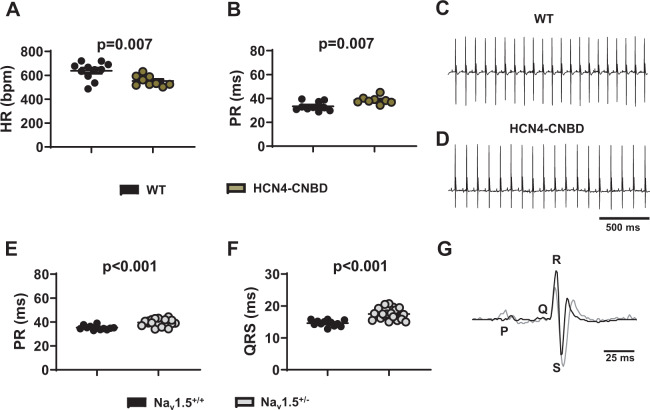
ECG characteristics of HCN4-CNBD and Na_v_1.5^+/−^ mice under basal conditions. 24 h heart rate and PR interval in WT (black circle) and HCN4-CNBD (green circle, **A**,**B**) together with the corresponding representative samples of telemetric ECG recordings (WT in **C**, HCN4-CNBD in **D**). 24 h averaged value of PR (**E**) and QRS (**F**) intervals recorded in WT (black circle) and Na_v_1.5^+/−^ (gray bars) mice. G: Close up of two superimposed ECG complexes from WT (black line) and Na_v_1.5^+/−^ (gray circle) mouse. Statistics: unpaired t test. Error bars define the s.e.m.

We also recorded HR in double transgenic mice expressing in a heart-specific and time-dependent manner a dominant negative HCN4 subunit devoid of sensitivity to cAMP (HCN4-CNBD)^18^. These mice express mutated HCN4 channels similar to patients presenting a familial primary form of SND^19,20^. Telemetric ECG recordings in these animals present with significant reduction in heart rate (552 ± 15 bpm, n = 9) and prolonged PR interval duration (39 ± 1 ms, n = 8), in comparison to control single-transgenic mice (Fig. 2A–D). We also measured the HR, the PR, and QRS intervals in mice haplo-insufficient for cardiac Na_v_1.5 Na^+^ channels (Na_v_1.5^+/−^)^27^. These mice recapitulate inherited forms of age-dependent progressive cardiac conduction-defective phenotype including progressive impairment of atrial and ventricular conduction^25,26^. As expected, we noticed a significant prolongation of both PR (40 ± 1 ms, n = 20) and QRS (17.5 ± 1 ms, n = 20) intervals in Na_v_1.5^+/−^ animals in comparison to WT (PR 35 ± 1 ms and QRS 14.5 ± 1 ms, n = 12; Fig. 2E–G). No differences were recorded in the HR between the two groups (533 ± 20 bpm and 531 ± 15 bpm, in WT and in mutants, respectively; Supplementary Fig. 3). Our data show that mouse models considered in this study are well designed to recapitulate a wide range of hallmarks of human primary SND, from moderate sinus bradycardia (HCN4-CNBD) to SND associated with severe AV block and atrial tachycardia (Ca_v_1.3^−/−^, Ca_v_1.3^−/−^/Ca_v_3.1^−/−^), as well as slowing of AV conduction (Na_v_1.5^+/−^).

### Inhibition of I_*KACh*_ by tertiapin-Q reduces SND and improves atrioventricular conduction in Ca_v_1.3^−/−^ and in Ca_v_1.3^−/−^/Ca_v_3.1^−/−^ mice

**Figure 3:**
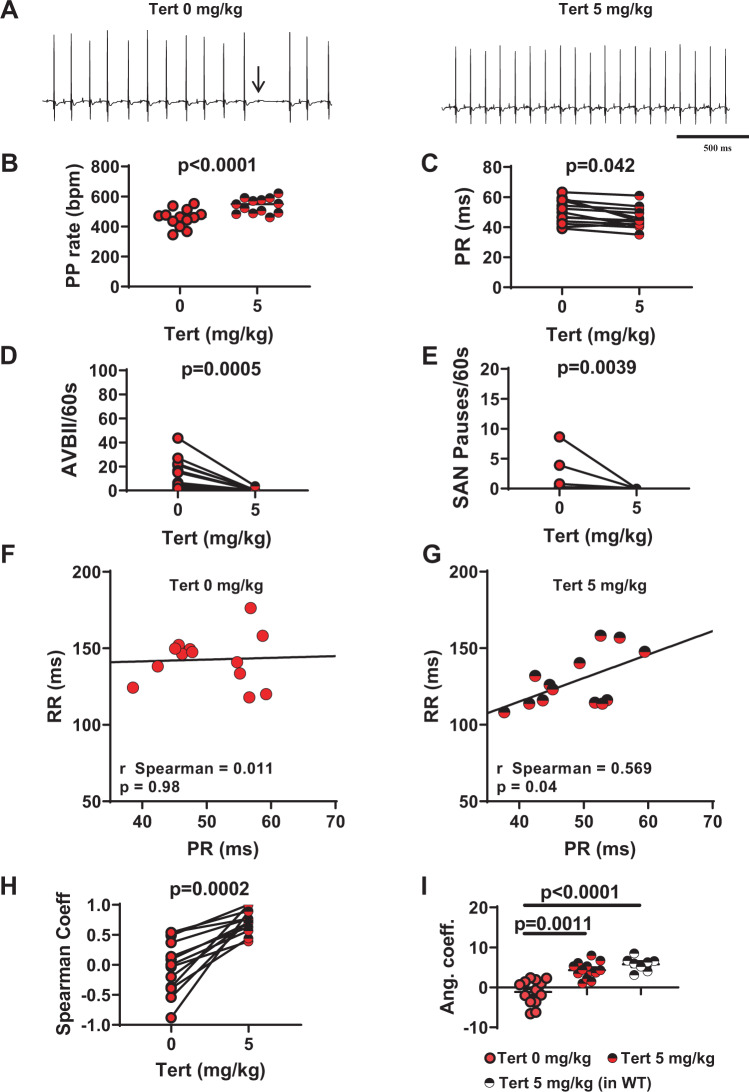
Tertiapin-Q effect in Ca_v_1.3^−/−^ SND mice. (**A**) Representative samples of telemetric ECG recordings from Ca_v_1.3^−/−^ mice before (left) and after (right) tertiapin-Q (Tert) injection; arrow indicates AVBII. B: PP rate increase following injection of tertiapin-Q. C: PR interval duration before (filled red circle) and after (horizontally red-striped circle) injection of 5 mg/kg tertiapin-Q. Statistics (**B,C**): paired t-test Number of AVBII (**D**) and SAN pause (**E**) counted in Ca_v_1.3^−/−^ animals in the absence (filled red circle) and in the presence (horizontally red-striped circle) of 5 mg/kg tertiapin-Q. Statistics (**D,E**): Wilcoxon test. Dot-plots of RR versus PR intervals measured in Ca_v_1.3^−/−^ mice, and relative linear regression line, in control conditions (**F**) and after injection of 5 mg/kg tertiapin-Q (**G**). (**H**) r-Spearman correlation coefficients calculated in control condition (full red circle) and after (horizontally red-striped circle) of 5 mg/kg tertiapin-Q. Statistics: Wilcoxon test. (**I**) Angular coefficients of the regression line between the RR and the respective PR intervals before (filled red circle), after injection of the peptide in Ca_v_1.3^−/−^ (horizontally red-striped bar) and after injection of the peptide in WT (horizontally black-striped circle) mice. Statistics: Kruskal-Wallis test followed by multiple comparison Dunn’s test. Error bars define the s.e.m.

**Figure 4:**
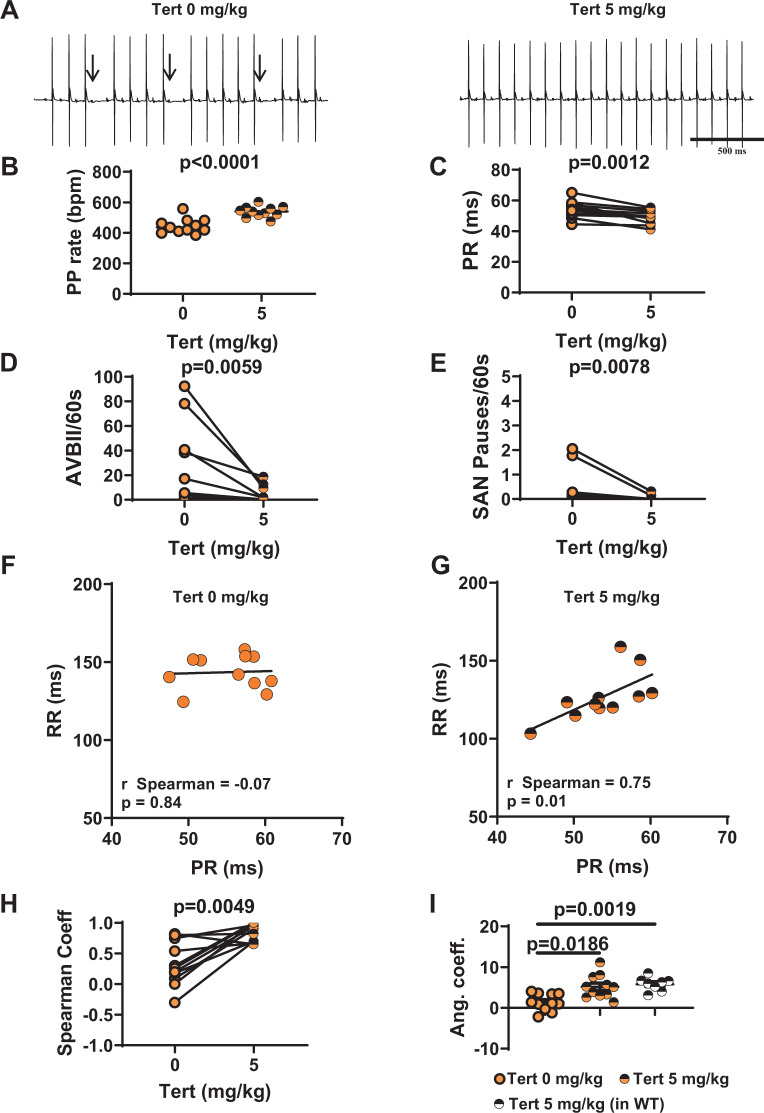
Tertiapin-Q effect in Ca_v_1.3^−/−^/Ca_v_3.1^−/−^ SND mice. (**A**) Representative samples of telemetric ECG recording in Ca_v_1.3^−/−^/Ca_v_3.1^−/−^ animals in absence (left) and in presence (right) of tertiapin-Q 5 mg/kg. Arrows indicate AVBII. (**B**) Effect of tertiapin-Q on PP rate in mutant mice. (**C**) Averaged value of PR interval before (filled orange circle) and after (horizontally orange-striped circle) injection of 5mg/kg tertiapin-Q. Statistics (**B,C**): paired t-test. AVBII (**D**) and SAN pauses (**E**) per minutes recorded in control conditions (filled orange circle) and after injection (horizontally red-striped circle) of the peptide. Statistics (**D**,**E**): Wilcoxon test. Ca_v_1.3^−/−^/Ca_v_3.1^−/−^ mice dot-plots of RR versus PR interval and relative linear regression lines in control condition (**F**) and in 5 mg/kg tertiapin-Q (**G**). (**H**) r-Spearman correlation coefficients calculated in control condition (filled orange circle) and after (horizontally orange-striped circle) of 5 mg/kg tertiapin-Q. Statistics: Wilcoxon test. (**I**) Angular coefficient values of the regression line of RR-PR intervals in control condition (full orange circle), after injection of the tertiapin-Q both in Ca_v_1.3^−/−^/Ca_v_3.1^−/−^ (horizontally orange-striped circle) and WT (horizontally gray-striped circle) mice. Statistics: Kruskal-Wallis test followed by multiple comparison Dunn’s test. Error bars define the s.e.m.

In a recent study, we showed that the G protein-gated *I*_*KACh*_ current constitutes a potential therapeutic target for the treatment of SND^29^. However, the effectiveness of *I*_*KACh*_ blockade in rescuing SAN and AV function in a wide set of models of primary SND *in vivo* has not been analysed. We, therefore, decided to investigate the effects of *I*_*KACh*_ inhibition by tertiapin-Q, a non-oxidisable analogue of tertiapin, on heart rate and AV conduction time in Ca_v_1.3^−/−^ and Ca_v_1.3^−/−^/Ca_v_3.1^−/−^ mice. In Ca_v_1.3^−/−^ animals, intraperitoneal injection of tertiapin-Q increased the heart rate in a dose-dependent manner, reaching a ‘plateau’ between 5 mg/kg and 10 mg/kg (Supplementary Fig. 4). At 5 mg/kg tertiapin-Q, the PP rate was increased by 18% (Fig. 3A,B) and the heart rate was increased by 28% (Supplementary Fig. 5A), the PR interval was shortened by about 10% (50 ± 2 ms and 45.5 ± 1 ms, n = 13, before and after injection, respectively; Fig. 3C), and the number of AVBII was drastically decreased (13 ± 4 AVBII/min before *vs*. 0.4 ± 0.3 AVBII/min after, n = 13; Fig. 3D). Finally, tertiapin-Q drastically reduced the incidence of SAN pauses (Fig. 3E) and the variability of PP interval (Supplementary Fig. 5B). In WT mice, a strong positive correlation was recorded between the PR interval and the ventricular rate (RR interval), either before (r_Spearman_ = 0.83, p < 0.05) or after (r_Spearman_ = 0.79, p < 0.05) injection of saline solution (Supplementary Fig. 6A–C). In Ca_v_1.3^−/−^ mice, we failed to observe a positive correlation between PR and RR intervals. However, injection of 5 mg/kg tertiapin-Q largely restored this positive correlation (r_Spearman_ = 0.57, p < 0.05; Fig. 3F–H). Moreover, administration of the peptide significantly increased the numerical value of the angular coefficient of the regression line between the RR and the respective PR intervals, underscoring its rescuing effect on AV function (Table 1; Fig. 3I). We then explored the effects of tertiapin-Q in mice lacking both Ca_v_1.3 and Ca_v_3.1 channels (Ca_v_1.3^−/−^/Ca_v_3.1^−/−^). To pursue this aim, we first performed a preliminary experiment on Ca_v_3.1^−/−^ mice, which show moderate SAN bradycardia^16^. Consistent with observations in Ca_v_1.3^−/−^ mice, injection of tertiapin-Q (5 mg/kg) rescued SAN bradycardia also in Ca_v_3.1^−/−^ mice (Supplementary Fig. 7), suggesting that tertiapin-Q could be effective in double-mutant mice. Consistent with this hypothesis, at a dose of 5 mg/kg, tertiapin-Q increased the PP rate by 21% (Fig. 4A,B) in these animals. AV conduction was severely compromised in Ca_v_1.3^−/−^/Ca_v_3.1^−/−^, as indicated by the significantly prolonged PR interval, the high number of AVBII per minute, and the absence of correlation between PR and RR intervals. Tertiapin-Q injection shortened the PR interval by 10% (55 ± 2 ms and 49.5 ± 1 ms, n = 11, before and after injection, respectively; Fig. 4C), strongly diminished the number of AVBII (25 ± 10 AVBII/min before vs 4 ± 2 AVBII/min after, n = 11; Fig. 4D), as well as the incidence of SAN pauses and the variability of PP intervals (Fig. 4E and Supplementary Fig. 8A). Moreover, tertiapin-Q restored the positive correlation between PR and RR intervals (Fig. 4F–H), and increased the numerical value of the angular coefficient of the RR-PR regression line (Table 2; Fig. 4I). Finally, tertiapin-Q dose-dependently increased the heart rate in Ca_v_1.3^−/−^/Ca_v_3.1^−/−^ mice (Supplementary Fig. 8B). Taken together, these data indicate that tertiapin-Q is able to ameliorate SND and to improve AV conduction in mouse models of Ca_v_1.3-dependent SND and congenital heart block.

**Table 1:** p-values of the angular coefficient of the RR-PR regression line calculated in Ca_v_1.3^−/−^ before (−1.0 ± 0.5) and after (7 ± 3) injection of 5 mg/kg tertiapin-Q and in WT animals after (6.0 ± 0.5) saline solution injection.

p value	Tert 0 mg/kg	Tert 5 mg/kg	WT
Tert 0 mg/kg		0.0006	0.0002
Tert 5 mg/kg			ns
WT			

(Kruskal-Wallis nonparametric test followed by multiple comparison Dunn’s test).

**Table 2:** p-values of the angular coefficients of the RR-PR regression line calculated in Ca_v_1.3^−/−^/Ca_v_3.1^−/−^ before (2.0 ± 0.5) and after (9 ± 3) injection of 5 mg/kg tertiapin-Q and in WT animals after (6.0 ± 0.5) saline solution injection.

p value	Tert 0 mg/kg	Tert 5 mg/kg	WT
Tert 0 mg/kg		0.0044	0.0204
Tert 5 mg/kg			ns
WT			

(Kruskal-Wallis nonparametric test followed by multiple comparison Dunn’s test).

### Effect of tertiapin-Q on SND caused by loss-of-function of f-(HCN) and Na_v_1.5 channels

**Figure 5:**
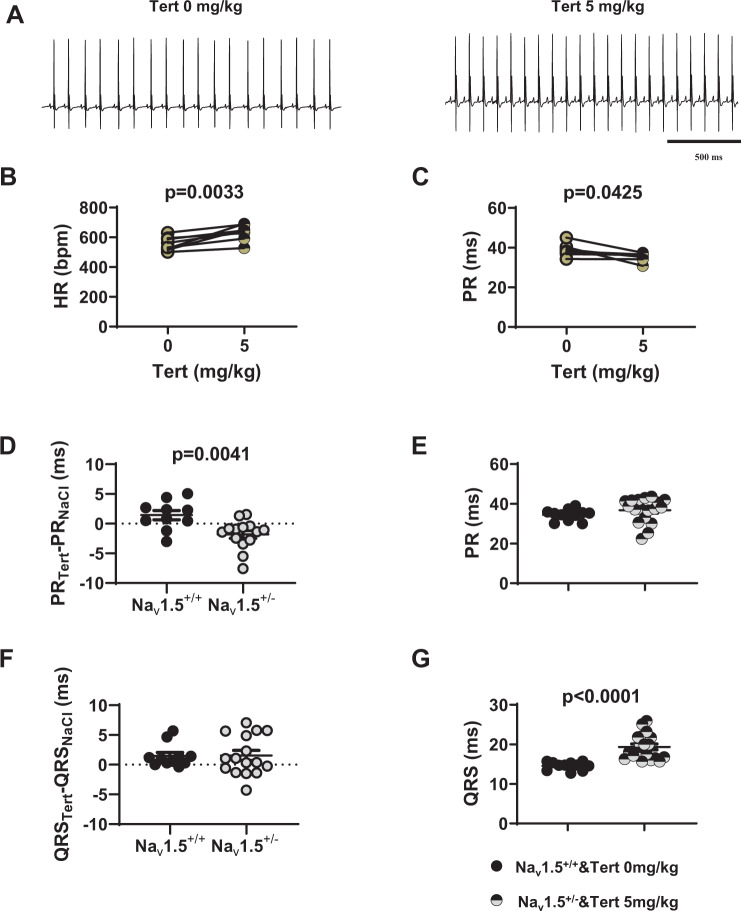
Effect of tertiapin-Q on heart rate and atrioventricular conduction in HCN4-CNBD and Na_v_1.5^+/−^ mice. (**A**) Representative samples of telemetric ECG recording HCN4-CNBD animals in absence (left) and in presence (right) of tertiapin-Q 5 mg/kg. Heart rate (**B**) and PR interval (**C**) in HCN4-CNBD mice before (filled light green circle) and after (horizontally green-striped circle) injection of 5 mg/kg tertiapin-Q. Statistics: paired t-test. (**D**) Values of the PR interval difference measured in tertiapin-Q 5 mg/kg and control (NaCl) condition in Na_v_1.5^+/+^ homozygous (Na_v_1.5^+/+^, black circle) and heterozygous (Na_v_1.5^+/−^, gray circle) mice. (**E**) PR intervals in Na_v_1.5^+/+^ mice in control condition (black circle) and in heterozygous mice injected with tertiapin-Q (horizontally gray-striped circle). (**F**) Values of the difference of QRS interval measured in tertiapin-Q 5 mg/kg and control (NaCl) condition in Na_v_1.5^+/+^ (black circle) and Na_v_1.5^+/−^ (gray circle) mice. (**G**) QRS interval in Na_v_1.5^+/+^ mice in control condition (black circle) and in heterozygous Na_v_1.5^+/−^ mice injected with tertiapin-Q (horizontally gray-striped circle). Statistics (**D**-**G**): unpaired t-test. *p < 0.05, **p < 0.01, ****p < 0.0001. Error bars define the s.e.m.

We then analysed the effects of I_*KACh*_ blockade by tertiapin-Q in HCN4-CNBD mice. At a dose of 5 mg/kg, tertiapin-Q totally restored the heart rate (556 ± 16 bpm before *vs* 633 ± 19 bpm after tertiapin Q) and the PR interval (38.5 ± 1.0 ms before *vs* 35.4 ± 0.5 ms after) in mutant mice to values similar to those recorded in control animals (Figs. 5A–C and 2A,B). In Na_v_1.5^+/−^ mice, PR and QRS intervals were significantly increased in comparison to control littermates (Na_v_1.5^+/+^, Fig. 2E–G). Tertiapin-Q injection significantly shortened the PR interval in Na_v_1.5^+/−^ animals (by about 2 ms, Fig. 5D). No significant difference was found between PR interval measured in Na_v_1.5^+/−^ animals injected with 5 mg/kg tertiapin-Q (36.5 ± 1.5 ms, n = 18) and Nav1.5^+/+^ mice injected with saline solution (34.5 ± 1.0 ms, n = 11; Fig. 5E). In contrast, tertiapin-Q did not significantly affect the QRS duration recorded in Na_v_1.5^+/+^ and Na_v_1.5^+/−^ mice (Fig. 5F). Indeed, the QRS interval in Na_v_1.5^+/−^ animals injected with 5mg/kg tertiapin-Q remained significantly longer than QRS measured in Na_v_1.5^+/+^ mice injected with saline solution (19.5 ± 0.5 ms, n = 18 in Na_v_1.5^+/−^ vs 14.5 ± 0.5 ms, n = 11 in Na_v_1.5^+/+^; Fig. 5G). We did not record any differences in heart rate between Na_v_1.5^+/−^ and Na_v_1.5^+/+^ animals as a result of the injection of the peptide (Supplementary Fig. 9A,B). Taken together, our data show that tertiapin-Q prevents SND and AV dysfunction caused by mutation of different ion channels involved in the generation and regulation of heart automaticity.

### Specificity of tertiapin-Q for I_KACh_

**Figure 6:**
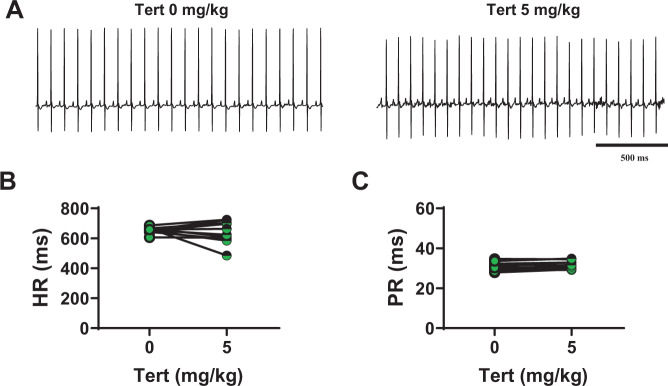
Tertiapin-Q effect on heart rate and PR interval in Girk4^−/−^ animals. (**A**) Representative samples of telemetric ECG recordings from Girk4^−/−^ mice before (left) and after (right) 5 mg/kg tertiapin-Q injection. Heart rate (**B**) and PR interval (**C**) in Girk4^−/−^ mice before (full green circle) and after (horizontally green-striped circle) injection of tertiapin-Q. Statistics: paired t-test.

To determine whether the effects of tertiapin-Q on HR and AV conduction in mutant mice was due to *I*_*KACh*_ inhibition, we studied the effect of this peptide in Girk4^−/−^ animals^36^. We previously showed that genetic ablation of GIRK4 channels leads to a complete loss of *I*_*KACh*_ in the SAN^37^. As expected, we did not observe significant effects of tertiapin-Q on heart rate in Girk4^−/−^ mice (at 5 mg/kg: 655 ± 6 bpm before injection vs 637 ± 22 bpm after injection, n = 11, Fig. 6A,B; Supplementary Fig. 10), or on the PR interval (31 ± 0.5 ms before injection vs 31 ± 0.5 ms after injection, n = 11, Fig. 6C). These results are in line with the hypothesis that the rescuing effect of tertiapin-Q on SND and AV conduction was due to inhibition of the cardiac GIRK1/GIRK4-channel complex mediating the cardiac *I*_*KACh*_.

### Comparison between different treatments used in clinics for acute SND and tertiapin-Q

**Figure 7:**
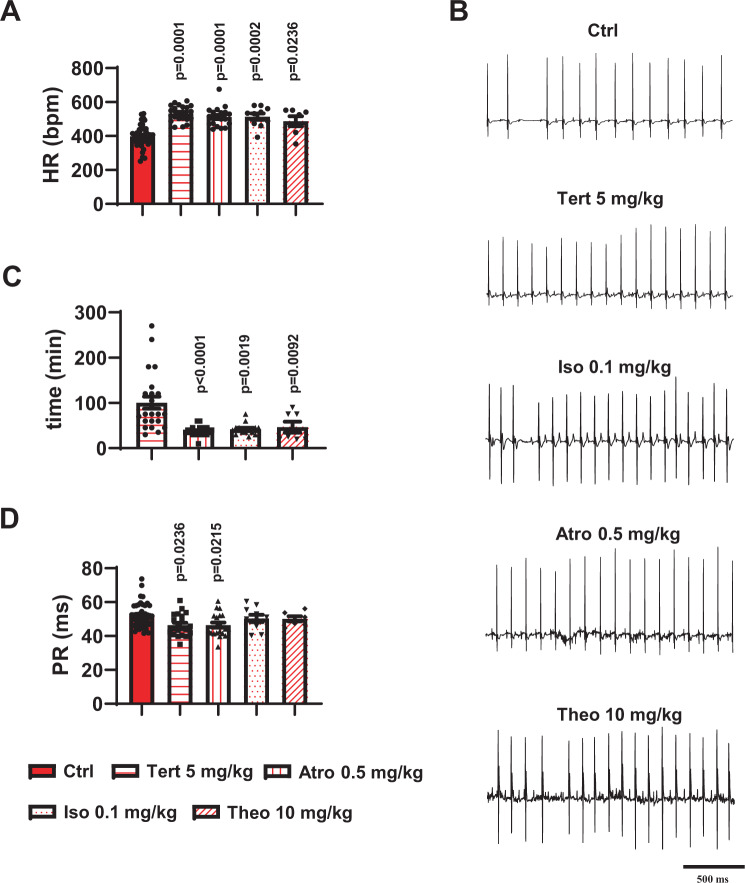
Effect of different molecules employed to treat bradycardia and cardiac conduction dysfunctions in comparison to tertiapin-Q in Ca_v_1.3^−/−^ mice. Heart rate (**A**) and corresponding representative ECG traces (**B**) recorded in Ca_v_1.3^−/−^ animals before and after injection of 5 mg/kg tertiapin-Q, 0.5 mg/kg atropine (Atro), 0.1 mg/kg isoproterenol (Iso) or 10mg/kg theophylline (Theo). (**C**) Duration of effect on the heart rate of the different molecules injected in Ca_v_1.3^−/−^ mice. (**D**) PR interval values measured in control condition and after injection of 5 mg/kg tertiapin-Q, 0.5 mg/kg atropine, 0.1 mg/kg isoproterenol or 10 mg/kg theophylline. Legend: control condition (red bar); 5 mg/kg tertiapin-Q (horizontally red-striped bar); 0.5 mg/kg atropine (vertically red-striped bar); 0.1 mg/kg isoproterenol (red-dotted bar); 10 mg/kg theophylline (transversally red-striped bar). Statistics: Kruskal-Wallis test followed by multiple comparison Dunn’s test. *p < 0.05; **p < 0.01, ***p < 0.001, ****p < 0.0001. Error bars define the s.e.m.

We sought to compare the effects of tertiapin-Q injection with those of other drugs that induce positive chronotropic effects and that are currently used in clinical practice to manage acute or transient SND^2^. To this end, we compared the effects of isoproterenol (Iso, 0.1 mg/kg), atropine (Atro, 0.5 mg/kg), or theophylline (Theo, 10 mg/kg) to those of tertiapin-Q (5 mg/kg) on HR and AV conduction parameters of Ca_v_1.3^−/−^ SND mice. In relation to AV conduction, we recorded the PR interval and the number of AVBII per minute to compare the capability of the different drugs to suppress hallmarks of AV dysfunction. The concentration of the different drugs was set according to the concentrations typically used for acute SND treatment in humans^2^. Tertiapin-Q, atropine, isoproterenol, and theophylline similarly increased the heart rate of Ca_v_1.3^−/−^ animals (Ctrl 399 ± 11 bpm, n = 37; Tert 5 mg/kg 533 ± 10 bpm, n = 21; Atro 0.5 mg/kg 512 ± 12 bpm, n = 20; Iso 0.1 mg/kg 514 ± 19 bpm, n = 10; Theo 10 mg/kg 487 ± 29 bpm, n = 7; Fig. 7A,B). The duration of the effect varied according to the drug injected. In Ca_v_1.3^−/−^ mice, the duration of tertiapin-Q effect was increased in a dose-dependent manner (Supplementary Fig. 11), reaching a maximal value at 5 mg/kg (100 ± 13 min, n = 23; Fig. 7C). Atro, Iso, and Theo had a shorter effect: 37 ± 3 min (n = 19), 42 ± 3 min (n = 14), and 46 ± 12 min (n = 7), respectively (Fig. 7C). We did not notice any difference in the effect of atropine and tertiapin-Q injection on PR interval duration, indicating a similar reduction in AV conduction time of the two compounds with respect to saline-injected Ca_v_1.3^−/−^ animals (Ctrl 52 ± 1 ms, n = 37; Tert 5 mg/kg 46 ± 1 ms, n = 21; Atro 0.5 mg/kg 46 ± 2 ms, n = 20; Fig. 7B,D). Strikingly, neither isoproterenol nor theophylline injection reduced the duration of the PR interval (50 ± 2 ms, n = 10 and 50 ± 1 ms, n = 7; Fig. 7D). Furthermore, to complete our data on AV conduction parameters, we examined whether Atro, Iso, or Theo could reduce the number of AVBII, as tertiapin-Q did, in Ca_v_1.3^−/−^ mice. Five mg/kg tertiapin-Q completely removed AVBII in 19 out of 24 animals tested (4 mice did not show AVBII under control condition; Table 3). Atropine eliminated AVBII in 14 out of 20 mutants (6 mice did not show AVBII under control condition; Table 3). On the contrary, both isoproterenol and theophylline injection did not significantly affect the number of AVBII. (Table 3). No induction of AVBII blocks following tertiapin-Q or atropine was observed in mutant mice that did not show AVBII blocks before injection. Taken together, these data suggest that inhibition of the effectors of muscarinic receptor pathway on the cardiac conduction system by tertiapin-Q or atropine improved, in addition to the HR, the AV conduction in Ca_v_1.3^−/−^ mice.

**Table 3:** Statistical analysis of AVBII reduction before (Pre) and after (Post) injection of tertiapin-Q (Tert), atropine (Atro), isoprenaline (Iso) and theophylline (Theo) in Ca_v_1.3^−/−^ mice.

	Tert 5 mg/kg (A)	Atro 0.5 mg/kg (B)	Iso mg/kg (C)	Theo 10 mg/kg (D)	p			
					A	B	C	D
Pre	20/4	14/6	11/3	4/4	**<0.0001**	**0.0256**	0.6776	>0.9999
Post	5/19	6/14	9/5	3/4				

Number of mice with AVBII/number of mice without AVBII; statistics: Fisher’s exact test.

## Discussion

Our work shows that inhibition of *I*_*KACh*_ by tertiapin-Q rescues SND and AV block to improve HR in model mice of primary SND induced by loss-of-function in ion channels involved in the generation of SAN pacemaker activity and AV conduction.

Furthermore, we found that contrary to isoproterenol and theophylline, tertiapin-Q concomitantly improves the HR and AV conduction. In contrast, while both isoproterenol and theophylline augment the HR, they fail to reduce the number of AV blocks. Our study indicates that tertiapin-Q could be a useful new candidate drug for the management of a wide range of primary SND forms, particularly for those associated with AV dysfunction.

### Mouse models of SND recapitulate hallmarks of SND in humans

We sought to explore the effects of tertiapin-Q on a wide range of mouse models recapitulating several different forms of human primary SND. To this end, we focused on models of SND induced by loss-of-function of ion channels involved in SAN pacemaker activity and AV conduction. Ca_v_1.3^−/−^ mice exhibit several hallmarks of SND, including SAN bradycardia and SAN pauses (Fig. 1)^29,38^. Atrial tachycardia and atrial fibrillation are also found in Ca_v_1.3^−/−^ mice upon intracardiac stimulation^24,29^. In recent studies on families with familial sinus node dysfunction and deafness (SANDD), it was reported that primary SAN bradycardia is associated with AV block due to loss-of-function of Ca_v_1.3^21,22^. SANDD hallmarks are similar to those observed in Ca_v_1.3^−/−^ mice, making this mouse strain particularly useful for modelling primary SND. We also included in our study Ca_v_1.3^−/−^/Ca_v_3.1^−/−^ mice to analyse the effects of tertiapin-Q on a model of congenital heart block (CHB). This autoimmune pathology affects the cardiac conduction system of foetuses and new-borns^39^. It is characterized by SAN bradycardia and progressive block of AV conduction. It has been shown that CHB is due to the production of autoantibodies against Ca_v_1.3 and Ca_v_3.1 leading to chronic inhibition of *I*_*CaL*_ and *I*_*CaT*_ in the conduction system of affected children^23,40^. In line with the proposed mechanism of CHB, Ca_v_1.3^−/−^/Ca_v_3.1^−/−^ mice recapitulate important hallmarks of CHB in relation to the pronounced SAN bradycardia, SAN pauses and severe AV block (Fig. 1)^17^. Here, we show that tertiapin-Q improves the SAN rate, reduces SAN pauses and effectively rescues cardiac conduction by eliminating AV blocks and restoring the physiological adaptation of the AV conduction time (PR). Overall, tertiapin-Q increased the HR (RR interval) of both Ca_v_1.3^−/−^ and Ca_v_1.3^−/−^/Ca_v_3.1^−/−^ mice in a dose-dependent manner by suppressing SND hallmarks and AV dysfunction.

In addition to VGCCs, several forms of primary SND due to loss-of-function of f-(HCN4)^19,20,41–43^ and Na_v_1.5 channels^44^ are known. In this study, we included HCN4-CNBD mice that constitute a model of primary HCN4-mediated SAN bradycardia due to the abolition of cAMP-dependent regulation of f-channels. Tertiapin-Q normalized HR and the AV conduction interval in these mice, which indicates that pharmacological inhibition of *I*_*KACh*_ could constitute an effective strategy for these forms of primary SND. More severe forms of primary SND due to loss-of-function of HCN4 channel conductance are known. In affected individuals, SND is associated with ventricular tachycardia and myocardial noncompaction^42,43^. Of particular note, we previously showed that genetic ablation of Girk4 prevents SND and associated arrhythmias in mice expressing dominant negative HCN4 channels with silenced channel conductance^45^, suggesting that inhibition of *I*_*KACh*_ could be an effective strategy also in severe forms of primary SND due to HCN4 loss-of-function. We employed Na_v_1.5^+/−^ mice to test the effects of tertiapin-Q in a model of AV dysfunction with reduced SAN bradycardia. While we recorded normalization of the AV conduction interval by tertiapin-Q, we failed to observe an effect of the QRS interval of Na_v_1.5^+/−^ mice. The reason for the absence of an effect of *I*_*KACh*_ inhibition on intraventricular conduction is unclear, but it could be due to the low expression of Girk4 in mouse ventricles^46^.

Studies on the genetic bases of primary bradycardia show that gain-of-function of *I*_*KACh*_ can cause SND^47–49^. The observation that an increased activity of Girk4 induces SND is consistent with the hypothesis that *I*_*KACh*_ activation may contribute to SND hallmarks in mouse models and humans and that pharmacological inhibition of this current could be an effective therapy.

### Comparison between tertiapin-Q and clinically used molecules for the management of bradycardia

Stimulation of β-adrenergic receptors by isoproterenol, inhibition of adenosine receptors by theophylline, or blockade of muscarinic receptors with atropine are currently used in clinical practice to treat acute SND^2,3^. The mechanisms of action of these drugs differ from mere *I*_*KACh*_ inhibition by tertiapin-Q. Isoproterenol increases the HR by elevating the intracellular concentration of cAMP in SAN pacemaker cells. This mechanism of action is, in part, similar to that of atropine, which increases heart rate by suppressing the antagonistic action of muscarinic receptors to cAMP synthesis, but also by preventing *I*_*KACh*_ activation. Finally, theophylline is expected to counteract adenosine-induced inhibition of cAMP synthesis and activation of GIRK1/GIRK4 K^+^ current (*I*_*Ado*_)^50^. In particular, activation of *I*_*Ado*_ is a major mechanism of SND secondary to heart failure^51^. However, in our conditions, while tertiapin-Q and atropine concomitantly normalized HR and rescued AV conduction, isoproterenol and theophylline failed to ameliorate AV conduction and to suppress AV blocks. These observations strongly suggest that direct inhibition of vagally-activated *I*_*KACh*_ is the critical factor contributing/leading to the rescue of heart automaticity as a whole in mouse models of primary SND not associated with heart failure. This view is in line with a previous study showing that high concentrations of ACh induce AV block in isolated guinea pig hearts, a phenomenon that can be prevented by perfusion of tertiapin-Q^34^. The precise reasons of why cAMP elevation and the consequent stimulation of ionic currents involved in AV conduction are not sufficient to prevent AV block are not fully understood. It is possible that the stimulation of inward currents facilitating impulse conduction through the AV node is not sufficient to overcome the negative dromotropic effect of *I*_*KACh*_ induced by the vagal tone. This possibility is consistent with our previous work showing that genetic ablation of Girk4 is required to maintain the equilibrium between inward and outward currents toward the inward direction in SAN pacemaker cells that are deficient in Ca_v_1.3 channels^29^.

### Conclusions and perspectives

In conclusion, our study shows that *I*_*KACh*_ inhibition by tertiapin-Q effectively rescues SAN bradycardia and associated AV dysfunction in mouse models of primary SND. In comparison to isoproterenol and theophylline, tertiapin-Q rescues both SAN bradycardia and AV block in these mice. Our data indicate that peptides derived from tertiapin-Q could be new promising cardio-specific drugs, with reduced side effects for pharmacological management of SND. Organic *I*_*KACh*_ blockers have been developed to treat atrial fibrillation^52^. Our study suggests that these blockers could be deemed potentially effective in managing also symptomatic SND.

## Materials and Methods

### Mouse models of SND

We have chosen mice (*Mus musculus*) as the model animal species for this study. Mice were considered for our experiments, because of the availability in our laboratory of genetically modified strains recapitulating congenital primary SND^53^. To the best of our knowledge, no alternative animal models of primary SND are available. Ca_v_1.3^−/−^, Ca_v_1.3^−/−^/Ca_v_3.1^−/−^, Girk4^−/−^, and double transgenic HCN4-CNBD mice were bred in a specific pathogen free (SPF) animal facility of the Institut de Génomique Fonctionnelle, Montpellier, France from C57B/6J genetic background. Na_v_1.5^+/−^ mice were generated in the SPF animal facility of the Institut du Thorax, Nantes, France from 129Sv genetic background. The investigation conforms to the Guide for the Care and Use of Laboratory Animals published by the US national Institute of Health (NIH Publication No. 85–23, revised 1996) and European directives (2010/63/EU). The experimental procedure was approved by the Ethical Committee of the University of Montpellier and the French Ministry of Agriculture (protocol n°: 2017010310594939). Animals (30 ± 1 g, sex ratio almost 50%) were housed in individual cages with free access to food and water and were exposed to 12-hour light/dark reverse cycles in a thermostatically controlled room.

### Telemetric recordings of ECG and analysis

For telemetric ECG recordings, adult mice (200 ± 10 day) were anesthetized with 2% isoflurane. A midline incision was made on the back along the spine to insert a telemetric transmitter, allowing simultaneous recording of ECG, body temperature and home-cage activity (ETA-F10, TA10EA-F21; DSI, St-Paul, NM, USA), into a subcutaneous pocket with paired wire electrodes placed over the thorax in DII derivation against the heart axis. Local anaesthesia was performed with 1% lidocaine injected subcutaneously at the sites of electrodes and transmitter implantation. Experiments were initiated after at least 15 days of recovery from surgical implantation. Mice were housed in individual cages with ad libitum access to food and water and were exposed to standard 12-h light–dark cycles in a thermostatically controlled room. ECG signals were continuously recorded (2000 Hz sampling rate) using a telemetry receiver and an analogue to digital conversion data acquisition system for display and analysis by Dataquest A.R.T. software (DSI, St-Paul, NM, USA). The SAN rate was determined from P-P intervals over 60 s time intervals. Heart rates (HR) were determined from ventricular inter-beat (RR) intervals of the ECG. Mean heart rate values were obtained in each mouse for an overall period of 24 hours. The instantaneous ventricular RR interval was used with the corresponding PR interval to evaluate the correlation between the RR and PR. For the evaluation of drug effects, the HR was first recorded for 4-h in baseline condition. Following drug intraperitoneal injection, mean heart rate values were calculated in each mouse by analysing periods of 5 min at different time points corresponding to the peak effect of the drug. Between two subsequent injections into the same animal, we at least waited 48 hours for total drug elimination from the organism. Atrioventricular (AVII) blocks and SAN pauses were counted by analysing a 1-minute interval every 15 minutes before drug injection and every 10 minutes during the drug effect. ECG parameters were measured with ECG Auto 1.5.7 (EMKA Technologies), or with Ponemah 5.20-SP7 (DSI, St. Paul, NM, USA) software. At the end of the protocol, animals were killed by cervical dislocation, according to the protocol approved by the ethical committee.

### Data and analysis

In order to achieve a statistical power of 0.90, the *a-priori* analysis calculation (G*Power 3.1) indicated to generate groups of 8-14 samples (depending on the parameters calculated in preliminary experiments). In all the experiments *n* indicates the number of independent values considered for the statistical analysis which was undertaken only for studies where each group size was at least n = 5. When group size, in preliminary experiment, was inferior to n = 5, no statistical analysis was applied and data were included in the Supplementary section. The use of transgenic mouse models generally prevented a real randomization (sex, age) in the generation of the groups and, in some cases, the equality in the size of the groups. All data were considered for the analysis. Statistical significance was defined for p values less than 0.05. In multi-group studies with parametric variables, post-hoc tests were conducted only if F value in the analysis of variance achieved the necessary level of statistical significance and there was no significant variance inhomogeneity. Otherwise, non-parametric statistical analysis was performed *ab-initio*. Blind analysis protocol was applied employing different operators for data recording and for data processing. Data, unless otherwise specified, are represented as mean ± SEM. Statistical tests used for each experiment were performed using Prism 6.0 (GraphPad Software) and are specified in the figure legends.

### Drugs

Tertiapin-Q was obtained from Smartox Biotechnology (Saint-Egrève, France) and from Alomone Labs (Jerusalem, Israel). Atropine sulphate (0.25 mg/ml) was obtained from Aguettant (Lyon, France). All others chemicals were obtained from Merck (Lyon, France).

## Supplementary information

Supplementary information.

## Data Availability

The datasets generated during and/or analysed during the current study are available from the corresponding author on reasonable request.
