# Supplementary material for: Inhibition of G protein-gated K+ channels by tertiapin-Q rescues sinus node dysfunction and atrioventricular conduction in mouse models of primary bradycardia
Source: Sci Rep. 2020 Jun 17;10:9835. doi: 10.1038/s41598-020-66673-8 (PMC7300035; doi:10.1038/s41598-020-66673-8)
Supplement: Supplementary file 1 — Supplementary information. [file 41598_2020_66673_MOESM1_ESM.pdf]

Title: Inhibition of G protein-gated K^+ channels by tertiapin-Q rescues sinus node dysfunction and atrioventricular conduction in mouse models of primary bradycardia

Running title: Pharmacological inhibition of I_{KACh} rescued sinus node dysfunction

Isabelle Bidaud[1,2]*, Antony Chung You Chong[1,2]*, Agnes Carcouet[3], Stephan De Waard[2,3], Flavien Charpentier[3,4], Michel Ronjat[2,3], Michel De Waard[2,3], Dirk Isbrandt[5,6], Kevin Wickman[7], Anne Vincent[1,2], Matteo E. Mangoni[1,2], @Pietro Mesirca[1,2]

[1]IGF, Univ. Montpellier, CNRS, INSERM, Montpellier, France.

[2]LabEx Ion Channels Science and Therapeutics (ICST).

[3]l'institut du thorax, INSERM, CNRS, UNIV Nantes, F-44007 Nantes, France.

[4]l'institut du thorax, CHU Nantes, Nantes, France

[5]Institute for Molecular and Behavioural Neuroscience, University of Cologne, 50937 Cologne, Germany.

[6]Experimental Neurophysiology, German Centre for Neurodegenerative Diseases (DZNE), 53175 Bonn, Germany.

[7]Department of Pharmacology, University of Minnesota, Minneapolis, USA.

* These authors contributed equally to this work

@ author for correspondence

Pietro Mesirca, PhD
Institut de Génomique Fonctionnelle
CNRS UMR 5203, Inserm U 1191
Université de Montpellier
141, rue de la Cardonille
34094 Montpellier, France
Phone +33 (0)4 34 35 92 46
Fax +33 (0)4 67 54 24 32
Email: Pietro.Mesirca@igf.cnrs.fr

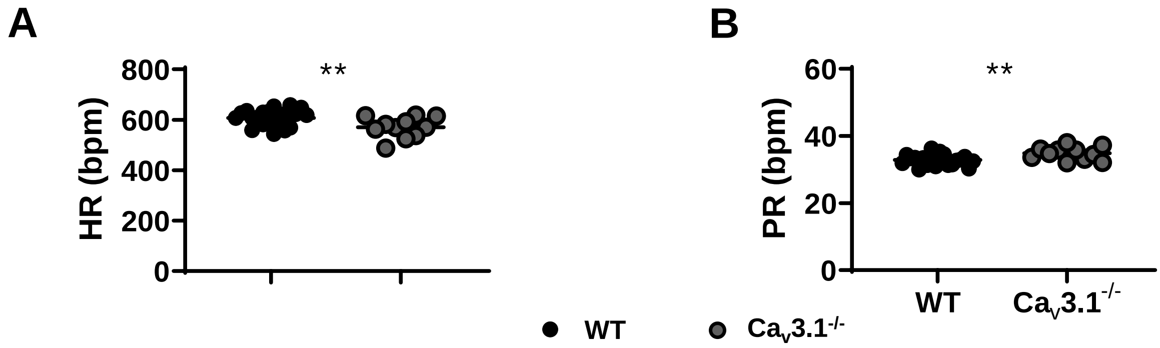

Supplementary figure 1. Heart rate and atrioventricular conduction time in $Ca_v3.1^{-/-}$ mice in basal condition. 24h heart rate (A) and PR Interval (B) recorded in WT (black circle, n=22) and $Ca_v3.1^{-/-}$ mice (gray circle, n =11). Statistics: unpaired t test. **p < 0.01. Error bars define the s.e.m.

SUPPL FIGURE 1

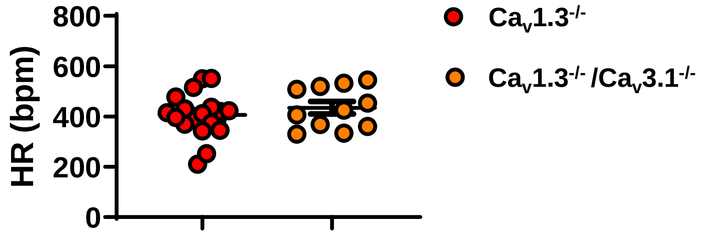

Supplementary figure 2. Heart rate in $Ca_v3.1^{-/-}$ and $Ca_v1.3^{-/-}$/$Ca_v3.1^{-/-}$ mice in basal condition. Heart rate recorded in $Ca_v1.3^{-/-}$ (red circle, n=18) and $Ca_v1.3^{-/-}$/$Ca_v3.1^{-/-}$ (orange circle, n=11). Statistics: unpaired t test. Error bars define the s.e.m.

SUPPL FIGURE 2

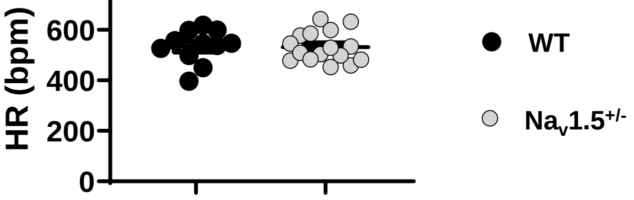

Supplementary figure 3. Heart rate $Na_v1.5^{+/-}$ animals in basal condition. 24h heart rate recorded in WT (black circle, n=11) and $Na_v1.5^{+/-}$ mice (gray circle, n=16). Statistics: unpaired t test. Statistics: unpaired t test. Error bars define the s.e.m.

SUPPL FIGURE 3

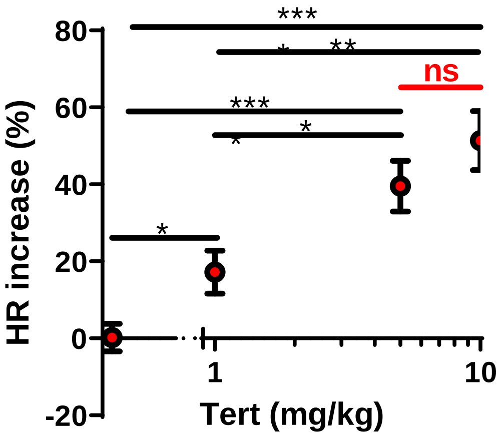

Supplementary figure 4. Dose response curve of heart rate in response to tertiapin-Q in $Ca_v1.3^{+/-}$ animals. Percentage of heart rate increasing in mutant $Ca_v1.3^{+/-}$ animals after injection of different doses of tertiapin-Q (n=11 at 0 mg/kg, n=11 at 1mg/kg, n=8 at 5mg/kg and n=6 at 10mg/kg); Statistics: analysis of variance (ANOVA) followed by Holm-Sidak multiple comparisons test. *p < 0.05, **p < 0.01, ****p < 0.0001. Error bars define the s.e.m.

SUPPL FIGURE 4

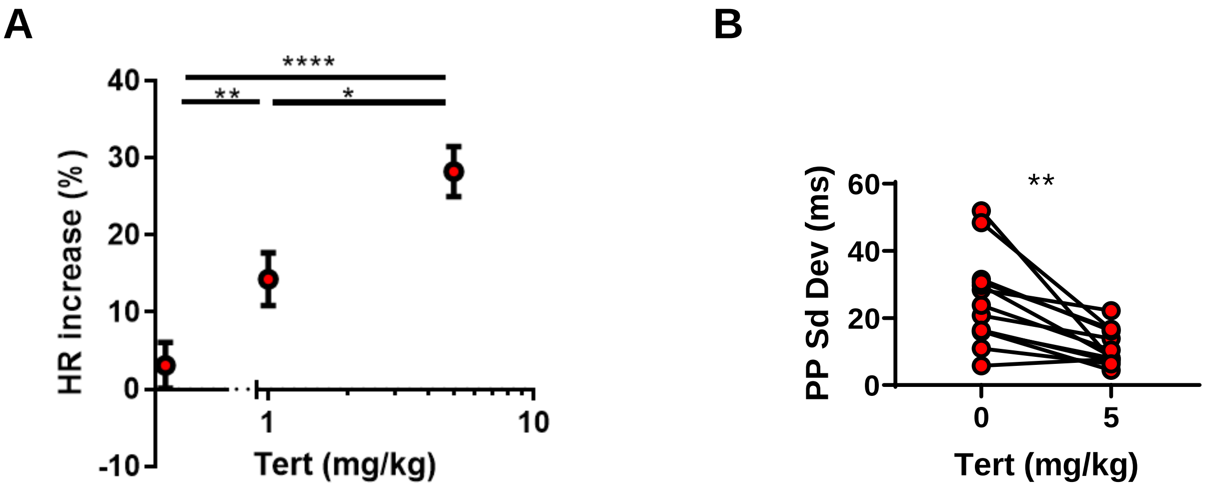

Supplementary figure 5. **A.** percentage of increase in the heart rate in response to injection of different doses of tertiapin-Q (n=19 at 0 mg/kg, n=24 at 1 mg/kg and n=23 at 5mg/kg) in $Ca_v1.3^{-/-}$ animals. Statistics: analysis of variance (ANOVA) followed by Holm-Sidak multiple comparisons test. **B.** PP interval variability before and after injection of 5 mg/kg tertiapin-Q (n=13). Statistics: paired-test. *p < 0.05, **p < 0.01, ****p < 0.0001. Error bars define the s.e.m..

SUPPL FIGURE 5

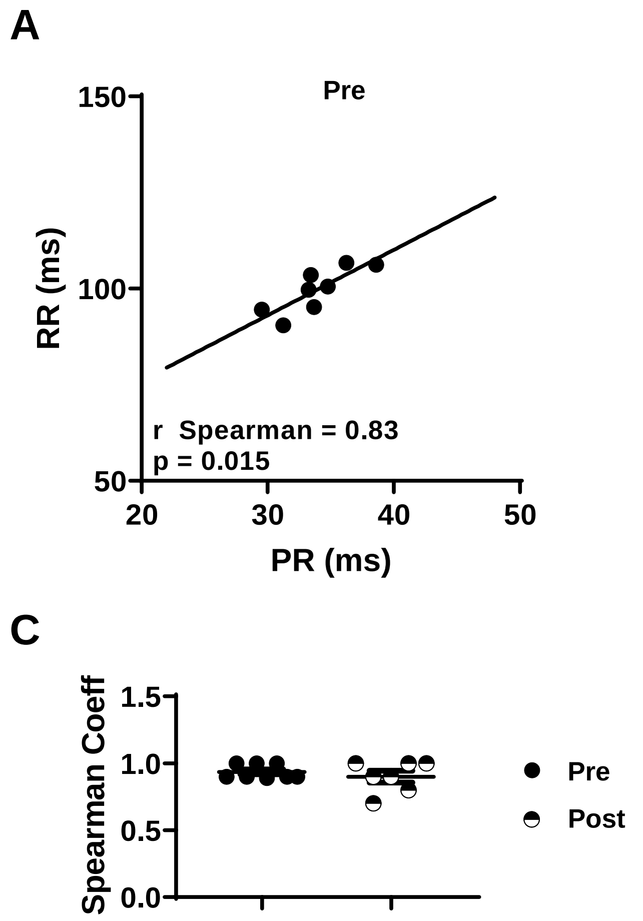

Supplementary figure 6. Effect of tertiapin-Q on RR-PR intervals correlation in WT animals.
Dot-plots of RR versus PR interval and relative linear regression lines in control condition (A) and in 5mg/kg tertiapin-Q (B) measured in WT mice (n=8). C: r-Spearman correlation coefficients calculated in control condition (full black circle) and after injection (horizontally black-striped circle) of 5mg/kg tertiapin-Q. Statistics: Mann-Whitney test. Error bars define the s.e.m.

SUPPL FIGURE 6

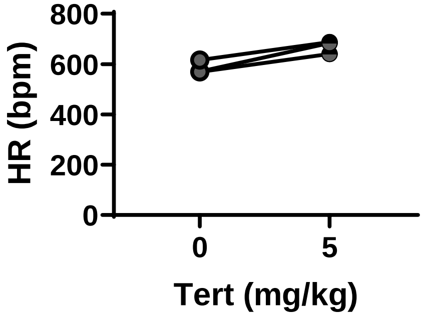

Supplementary figure 7. Tertiapin-Q effect on heart rate in $Ca_v3.1^{-/-}$ mice.
Heart rate measured before (full gray circle) and after (horizontally gray-striped circle) injection of 5mg/kg tertiapin-Q in $Ca_v3.1^{-/-}$ animals (n=3).

SUPPL FIGURE 7

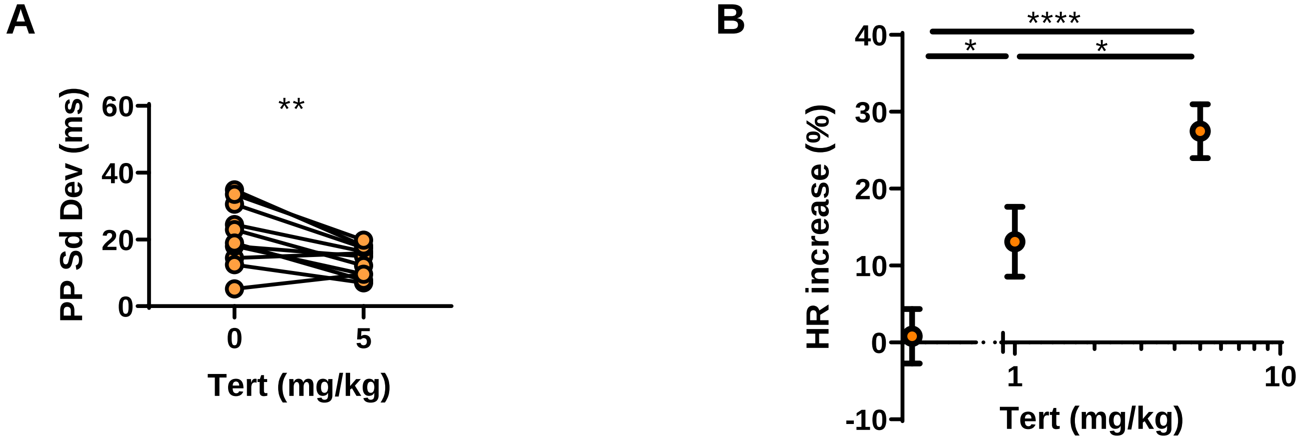

Supplementary figure 8. A. PP variability before and after 5mg/kg tertiapin-Q injection in $Ca_v1.3^{-/-}/Ca_v3.1^{-/-}$ mice (n=11). Statistics: paired-t-test. B. percentage of increase in the heart rate in response to injection of different doses of tertiapin-Q (n=11 at 0 mg/kg, n=10 at 1 mg/kg and n=11 at 5mg/kg) in double-mutant animals. Statistics: analysis of variance (ANOVA) followed by Holm-Sidak multiple comparisons test. *$p < 0.05$, **$p < 0.01$, ****$p < 0.0001$. Error bars define the s.e.m..

SUPPL FIGURE 8

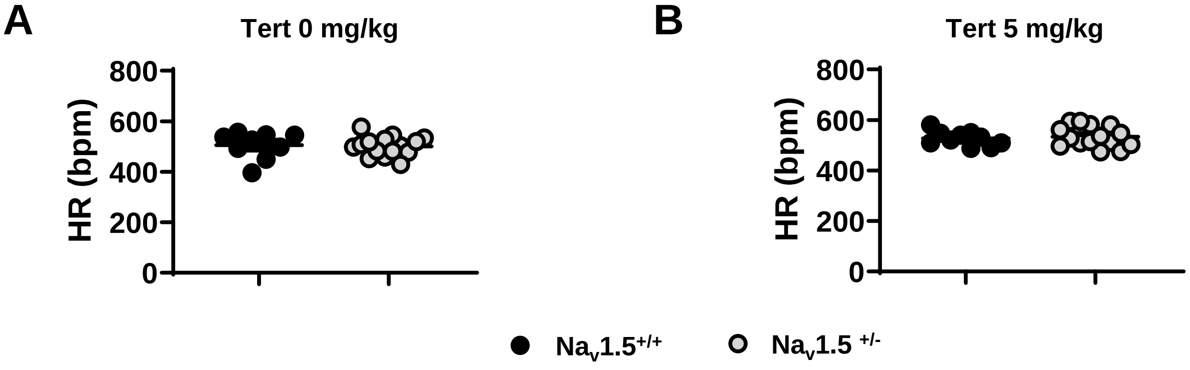

Supplementary figure 9. Tertiapin-Q effect on heart rate in $Na_v1.5^{+/-}$ mice.
Heart rate measured in 5mg/kg tertiapin-Q and control (NaCl) conditions in $Na_v1.5$ homozygous (black circle, n=9) and heterozygous (gray circle, n=15) animals. Statistics: unpaired t test. Error bars define the s.e.m.

SUPPL FIGURE 9

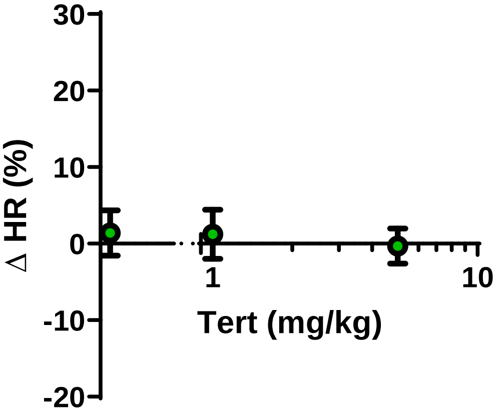

Supplementary figure 10. Dose response relationship of heart rate in response to tertiapin-Q injection in Girk4$^{-/-}$ animals. Percentage of heart rate increasing in mutant Girk4$^{-/-}$ animals after injection of different doses (n=6 in control condition, n=6 at 1 mg/kg and n=10 at 5 mg/kg) of tertiapin-Q; Statistics: analysis of variance (ANOVA) followed by Holm-Sidak multiple comparisons test. Error bars define the s.e.m.

SUPPL FIGURE 10

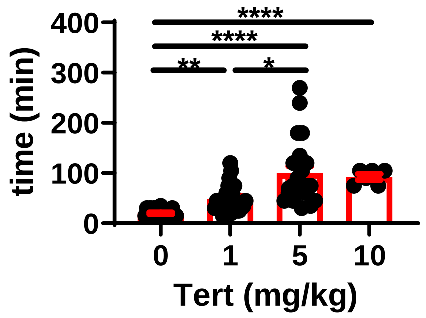

Supplementary figure 11. Dose response curve of the duration of the 'rescuing' effect on heart rate in response to tertaipin-Q injection in $Ca_v1.3^{-/-}$ animals. Duration of effect on the heart rate after injection of different doses of tertiapin-Q in $Ca_v1.3^{-/-}$ mice (n=19 at 0 mg/kg, n=24 at 1 mg/kg, n=23 at 5 mg/kg and n=6 at 10 mg/kg). Statistics: Kruskal-Wallis test followed by multiple comparisons test. *p < 0.05, **p < 0.01, ****p < 0.0001. Error bars define the s.e.m.

SUPPL FIGURE 11
